# Surgery

**Published:** 1837-10

**Authors:** 


### SURGERY.

### *On the Relations and Proportions of the Muscles of the Right and Left Sides in Lateral Curvature of the Spine.* By Dr. GUNTHER, of Hamburg.

IN all cases of lateral curvature of the upper dorsal vertebræ, there exists a spot more developed, or full, near the spinous processes of the lumbar vertebræ of the opposite side. This has led to a general belief that the muscles of the one side are, in their form, development, and power, very different from the corresponding muscles of the opposite side. A circumstance which has further strengthened this opinion is, that, where the curvature has been very considerable, the ribs of one side are much more nearly approximated, and the interval between the last rib and crest of the ilium much less, than on the opposite. The external configuration of the muscles on the two sides is also very dissimilar.

Authors have not paid sufficient attention to the point in question by dissection of the muscles; and even Jörg has neglected to examine their condition in this malformation, although he has given such an accurate description of the altered relations of the bones. In accordance with the opinion of Jörg, Maisonabe maintains that the muscles of the convex side become lengthened and discoloured, and undergo a change of structure approaching to cellular tissue. Shaw, who appears to give the result of his own dissections, denies this, and states that the muscles of the concave side are not particularly contracted, nor those of the convex side remarkable for their wasted appearance. Heidenreich believes the full appearance opposite the curvature to be, in the first place, rather the result of increased strength and development of the muscles than of the altered configuration of the skeleton. Delpech surmises, without giving his opinion as the result of his dissections, that all the muscles of the back are thin, pale, and weak; but he does not think that the muscles of one side are commonly more developed than those of the other.

Some years since an opportunity offered itself to Dr. Günther of examining the body of a full-grown adult who was the subject of distortion of the spine; and, although he was not then able to devote much time to the dissection, he remarked that the appearances presented by no means tallied with the accounts generally given. The muscles in this case were equally red on either side, and of about the same density: and, even the intercostal muscles of the side on which the ribs were actually overlapping one another, were by no means shrunk or wasted, as described by Jörg. A few months before the present report was written, Dr. Günther had another opportunity of examining an adult subject similarly circumstanced; and although, from the necessity of deferring the dissection, he was obliged to place the body in spirit for a short time, which to a certain amount interfered with the colour and consistence of the muscles, he was enabled to make some accurate observations. The following are the general results at which he has arrived:—1. All the muscles which act either directly or indirectly on the spinal column continued well developed: no one in particular was shrunk. 2. The form and figure of the muscles, whilst unseparated from their attachments, were much altered; as, for instance, the latissimus dorsi, rhomboidei, trapezius, &c.; their elevation and consequent extension upwards on one side being accompanied by an opposite condition on the other; but, when removed from the body, these same muscles corresponded both in length and breadth. 3. It thus becomes intelligible that the muscles which occupy the hollows are not really shortened or shrunk, but assume a folded form; whilst those which are connected to the corresponding arches are not uncommonly stretched, and the osseous frame-work is likewise compelled to accommodate itself to the soft parts. 4. The object the muscles have to accomplish is, to preserve the equilibrium, *i. e.* so to act as to retain the occipital protuberance in a line vertical to the middle ridge of the sacrum: thus, the head in these individuals rarely deviates from the perpendicular, the distorting effect of one curvature being always compensated for by another; and, in the end, the shoulders are nearly always of the same height. In accordance with these principles, it will be perceived that all the muscles acting on the spine have, as nearly as may be, an equal share of weight to bear on the right and on the left side; a fact which satisfactorily accounts for the equality of development as modified by circumstances to be presently noticed. 5. A remarkable contrast may, however, be observed if individual corresponding muscles are compared; and this difference is doubtless regulated by the same principle, that of dividing the weight and labour equally.

From the foregoing premises the following deductions may be made; the results depending on the variations of power and weight, and the bulk and altered attachments of the muscles:—1. As soon as muscular power operates under unfavorable circumstances on one side, in consequence of displacement or distortion of the bony frame-work, the muscles so circumstanced gain in mass and power. Such unfavorable conditions are, for instance, when the arm of the lever to be moved is shorter, or the angle of attachment of the muscle has become more acute; or, further, if the muscle itself has, from position, become folded in such a manner as to require very vigorous contraction to act upon its point of insertion. 2. If the connexions of a muscle become so unfavorable that it can no longer act at all on its normal points of attachment, or at best but very imperfectly and inconveniently, (especially where the plaiting or folding is so considerable as to produce these disadvantageous results;) such muscle will then change its points of insertion, either by separating itself from one spot and attaching itself to another and more convenient point, or it will derive a fresh origin by newly developed heads.

In order the more accurately to ascertain the deviations of the spinal column from the perpendicular, a large pin was struck into the spinous process of each vertebra of the body of a moderately muscular man, forty-five years of age, who, long before death, had laboured under a sigmoid curvature of the spine. A straight line being drawn from the superior occipital protuberance to the middle of the sacrum, it was observed that the first four cervical vertebræ corresponded to this line, with the exception of a slight inclination of the atlas and axis to the left. From the fifth cervical to the seventh dorsal vertebra, a curvature extended to the left; the centre of the arch or extreme deviation from the perpendicular (about fourteen Parisian lines) being opposite the fourth dorsal. The vertical line then intersected the space between the seventh and eighth dorsal vertebræ, at which point the curvature to the right commenced. This second arch reached as low as the third lumbar vertebra, and thus comprehended eight vertebræ, the three lowest dorsal being farthest removed (ten Parisian lines,) from the perpendicular. The fourth and fifth lumbar vertebræ were then intersected by the vertical line. By this distortion, to which may be added a curvature forwards in the lumbar region, this man had lost nearly three inches of his height. The upper ribs of the right side were much arched backwards, whilst the corresponding ribs of the opposite side were approximated and flattened. The inferior angle of the right scapula was an inch and a half below that of the left; but the acromion process was, as is usually the case, of the same height on both sides.

The following are a few of the changes observed in the muscular system; and they illustrate the principles laid down:—1. The *trapezius* of the right side was greatly extended, partly by the arching of the ribs, in part because of the depressed position of the scapula and its abnormal distance from the vertebral column. The muscle of the left side was, on the contrary, thrown into plaits; the consequence of which arrangement was, that the right muscle could produce with facility the same results which required vigorous contraction of the left to effect. A comparison of the two muscles bore out this position; for the left was the redder, and proved to be by far the heavier of the two. Nevertheless, when removed from the body and spread out, the two muscles corresponded precisely in length and breadth. 2. The *latissimus dorsi* was likewise much extended on the right side, covering the inferior angle of the scapula to the extent of two inches, and arching over the prominence of the vaulted ribs; whilst the left was folded and gave no covering to the scapula. The upper margin of the right muscle arose at a right angle from the vertebræ; but the left took its origin at an angle the acuteness of which increased more and more as low down as the last dorsal vertebra: the latter also far exceeded the former in weight, although, when spread out, their superficies was the same. 3. The *recti* muscles of the abdomen further partook of the modification resulting from the altered form of the skeleton: that of the right side was thin, weak, and interwoven with cellular and adipose tissue, and not more than a sixth part of the weight of the left.

The other muscles noticed are adduced as evidence that various means are had recourse to by nature for equalizing power under unfavorable circumstances, independently of the simple increase of structure; these means consist in a complete or partial transfer of the muscular attachments from the original to some new and more advantageous position. 4. In consequence of the altered situation of the scapulæ, the relation of the *rhomboidei* and *levatores scapulæ* muscles varied much on the two sides. The left rhomboideus (we name the two as one,) was acting, like the trapezius, under great disadvantage: it was not, however, shortened in consequence of the approximation of its origin and insertion, but lay folded beneath the scapula: but in this position it could not act upon its line of insertion: how, therefore, was this difficulty remedied? The whole muscle was found to have become nearly entirely detached from the base of the scapula, and had contracted a firm and strong attachment solely to its inferior angle; thus assuming the form of a single-penniform muscle. Now, the action of this muscle with such an attachment must have been necessarily limited to the elevation of the inferior angle of the scapula, an effect which, as not desirable, was compensated for by the arrangement of the *levator scapulæ*. This muscle, in consequence of the abnormally elevated position of the left scapula, would have been inert on this side, had it not been furnished with two extra heads; accordingly, beside its regular points of origin, an additional head arose from the oblique process of the fifth cervical vertebra; and another very strong one from the spinous process of the fourth. These two heads were tense, but the normal heads were relaxed; and thus the combined action of this and the rhomboid muscles was in the end rendered similar to that of the corresponding muscles on the opposite side. In accordance with the analogous condition of the other muscles, the left rhomboid and levator of the scapula were much heavier than those of the right. 5. Lastly, the action of the *omo-hyoid* muscles was necessarily interfered with by the altered position of the scapula. In consequence of the bulging backwards of the ribs on the right side, the corresponding scapula was naturally carried back likewise; whilst the left scapula, as will be remembered, was approximated to the median line. The cellular connexion between the omo and sterno-hyoid muscles, together with the tendinous intersection of the former, existed as usual; only its scapular attachment was in part wanting. The right omo-hyoid was connected to the acromial half of the clavicle, along the line where it usually lies under cover of this bone; and its scapular attachment had degenerated into a mere process of condensed cellular tissue. A similar conversion had commenced on the opposite side, but was not completed; for the left muscle was attached by an independent head to the collar-bone, whilst the normal origin from the scapula was weak and partly converted into tendon.

It will naturally be asked whether these observations are likely to assist us at all in the treatment of the disease in question. We will only here throw out the following hints:—1. The plan of rubbing spirituous embrocations on the convex, and oily embrocations on the concave side, under the impression that increased vigour will result from the former method, and relaxation from the latter, are both grounded on false principles, inasmuch as no absolute contraction of the muscles exists; and their relative power is not directly dependent upon the form of the osseous framework, but regulated by the laws for equalizing the distribution of weight. 2. The advice to strengthen certain muscles on one side by specified exercises cannot be correct, because the evidence which dissection has afforded, proves that there is an ample compensation for the loss of advantageous attachment, by increase of bulk of the muscles so circumstanced: thus, the evil would by this plan of treatment be augmented instead of diminished. 3. As all muscles which assist in maintaining the perpendicularity of the vertebral column in its normal condition, act by tending to approximate the head and each vertebra to the sacrum, so can aid only be expected as a consequence of increased strength where the natural form of the spine has been previously restored, or where extension of the column is combined with exercise of the muscles. Nevertheless, any means of giving tone to the muscles, if employed with careful attention to the general condition of the patient, cannot but operate beneficially in improving the general health. 4. In cases of long standing, where there is an absolute transfer of the attachments of muscles, of course a return to the normal condition and a radical cure are out of the question.

*Pfaff's Mittheilungen, &c.* Heft ix.-x. 1836.

---

*On Suture of the Intestine.* By M. Fleury.

M. Fleury, in a Memoir on the Intestinal Suture, relates three cases in which M. Jobert's method of closing wounds of the intestinal canal was put in practice. This method is founded upon experiments, the results of which may thus be stated.

1. If a ligature be applied on healthy intestine, it acts in the same manner as on an artery, cutting the mucous and muscular coats, whilst the serous coat resists its action. 2. If a ligature be applied on a portion of intestine, when the serous membrane is inflamed, all three coats are divided, even though the knot is tied with the least possible force. 3. If two serous surfaces are placed in contact, and the contact slightly maintained, agglutination takes place at the end of an hour; a result which need not surprise us, when we consider the rapidity with which false membranes and adhesions are formed in inflammation of serous tissues.

The operation then consists in bringing the two serous surfaces in contact, by means of a ligature which is not tied, but gently twisted, and which may be withdrawn at the end of a few days.

In the first two cases related by M. Fleury, the result was successful, *quoad* the immediate object of the operation, although from other causes both terminated fatally. The third case was more fortunate.

A lady, aged fifty-four, had had crural hernia on the left side for many years, which had occasionally been strangulated, but had been always reduced by the taxis. It again became strangulated on the 25th November, 1836, and after many ineffectual attempts at reduction, the operation was resolved on. The intestine had now been strangulated fifty hours. The usual incisions were made, and the sac being opened, M. Jobert was astonished to find that it contained nothing but a straw-coloured serum, and terminated in a cul de sac. Further examination convinced him that this was an old obliterated sac, and that below it existed another containing strangulated intestine. Accordingly, a further incision opened the true hernial sac, which contained several loops of intestine, much distended and adherent to each other. By accident, the bistoury penetrated the intestine, and a quantity of gaseous and fæcal matter escaped, to the great relief of the patient. The stricture having been divided, the operator determined to close the wound in the intestine by suture. A common needle was introduced into the intestine from without about three or four lines below the edge of the wound, and brought out about half a line from the edge; it was then introduced on the opposite lip of the wound, and carried from within outwards, so as to emerge about three or four lines from the edge. The threads being then brought together, gentle torsion was applied, which brought together the external edges of the wound, and placed the serous surfaces in contact. The intestine was then returned into the abdomen, and the threads being retained without by adhesive plaster, the ordinary dressings were applied. The symptoms were from this time favorable; on the fourth day the bowels acted well; on the sixth day the wound was dressed, and one of the threads withdrawn; on the eighth day the second ligature was removed, and the wound healed rapidly. Three months after the operation, the patient was quite well, the bowels acting regularly.

[A comparison of this case with that of Professor Dieffenbach's related in the April Number of this Review will show the superiority of M. Jobert's method. In Dieffenbach's case the patient survived several weeks after the operation, and had returned to his usual occupations; yet on examination, suppuration was found to be still going on round the ligatures which had been cut off close, and left within the abdomen. It will therefore be advisable to use torsion in preference to tying a knot, even should the intestine be in a perfectly healthy state.]

*Archives Générales de Médecine. Mars,* 1837.

---

¶ *On the Nature and Treatment of Itch.*

[THE following articles, extracted from different Journals, we have placed together for greater facility of comparison and contrast. The subject is trite, but not the less important on that account.]

I. *On the Influence of the Human Acarus in the Production of Itch.* By M. ALBIN GRAS.

SEVERAL eminent physicians, and among others M. Rayer, (in the last edition of his Treatise on Cutaneous Diseases,) having doubted if the acarus be really the cause of the itch, M. Gras proposes, in the present memoir, to answer the three following questions:

1st. Is the acarus always observed in this disease, and never apart from it?

M. G. has never found the insect elsewhere than in cases of itch. On examining the hands only of those who *first* presented themselves for treatment, the insect was observed in nine out of ten cases: the exceptional instances he notices might have presented it in other parts of the body, or the patients might have been under treatment furtively.

2d. Is the acarus the agent of the contagion of itch?

To resolve this question, he states that, independently of successful inoculation on his own person, he placed some acari between the fingers of a fellow student, who was fairly infected, although all contact with other patients had been avoided; and, of two young women who had the acari placed in the arm-pit, one (and one only) took the disease, and communicated it to her father and mother. On the other hand, his own experiments, and those of MM. Mouronval and Lugol, in 1821, have proved the futility of inoculation from the vesicles of the itch. As a further confirmation, he adds that, after two or three applications of sulphur ointment, all the acari discovered were dead, although the eruption, and even the formation of new vesicles, continued. Daily experience attests that the disease ceases to be contagious after two or three days' treatment. The acarus appears to possess a complicated organization: it lays eggs; and the copulation of two allied species, the acarus of the sheep and that of the horse, has been observed; it is, therefore, too perfect to be the produce of spontaneous generation, even if this theory were admissible. Were the acarus not the cause of the contagion, the itch should be found without it, a fact at least very rare; or the acarus should exist without the itch, which has not yet been noticed.

3d Question. In what way does the insect produce the itch; by direct and mechanical irritation, or by means of a peculiar virus?

On examining the vesicles of the hand, some few are observed to be situated at the origin or by the side of the little grooves hollowed by the insect, but by far the greater number exhibit no trace of its presence: on the contrary, fifty or more insects were sometimes discovered on the hands when there were few vesicles, and hardly any pruritus. These animals are with certainty destroyed by a few inunctions, but fresh crops of distinct and acuminated vesicles will often continue to arise for a fortnight or more. For these and other reasons, M. Gras considers that the acarus exerts upon the skin a physiological and vital action, by virtue of a particular virus; using the word virus as synonymous with the phrase, "unknown agent, producing great effects by a trifling visible action." He compares the *modus operandi* to that of certain insects, which, placing their little egg upon the oak or other vegetable, develop, each after its kind, numerous varieties of excrescences.

*Journal des Connaissances Médicales. December,* 1836.

II. *On the Nature and Treatment of Itch.* By Dr. Pentzlin, of Wismar.

The cause of itch still remains involved in some obscurity. The modern French school considers the Acarus scabiei as the sole cause of its propagation, and refuses to allow contagious properties to the contents of the vesicles or pustules. Dr. Pentzlin, of Wismar, is disposed to consider this view of the subject as erroneous, and he regards the insect as a mere parasite, which owes its existence to "generatio æquivoca." Experiments have been adduced to prove that the disease can be communicated by means of the insect only; but Dr. Pentzlin, in such cases, ascribes the contagious property, not to the insect itself, but to adhering virus.

It has been shown by statistical papers, that about thirty-four days are required for the cure of itch in the hospital of the Charité at Berlin; in the hospital of Wismar four, or at most seven, days suffice. In the latter the use of sulphur has been renounced, and an ointment is now adopted, composed of one part of tar and two parts of salt butter, to which, when melted, one part of common potashes is added. The patient is stripped and put to bed, and frictions with the above ointment, repeated every twenty-four hours, are applied over the whole body. Four, or at most seven, applications suffice, and the treatment is finished by a warm bath.

*Gräfe and Walther's Journal.* Vol. xxiv. 1836.

III. *Comparison of the Results from the homœopathic and the common Treatment of the Itch.* By Dr. Klein, Army Surgeon at Stuttgardt.

By command of the government, twenty-eight recruits with the itch were treated, fourteen of them by an homœopathist, Dr. Steinestel, and fourteen in the usual way, by Dr. Klein. The homœopathist, who had at his disposition all the remedies which his system required, gave to his patients, according to his own account, infinitesimal doses of calcaria, sulphur, and the scabietic matter itself; ordered them to be washed with soap and warm water daily; to take a bath frequently, and afterwards, as summer came on, to bathe every day in the Neckar, having rubbed in, previously, all over the body, a portion of sulphurous soap. The results of this treatment, which, whether it was purely homœopathic or not we leave to our readers to decide, were as follows:

| | | | |
|---|---|---|---|
| 6 men were dismissed cured in | | | 10 weeks, |
| 4 do. | — | — | 13 do. |
| 1 do. | — | — | 19 do.; |

of these, two suffered a relapse, or were newly infected in a few days, though the homœopathist had previously denied that either of these events could occur. After having been under treatment for twenty weeks, the remaining three were neither cured nor improved. At this period, the commander of the regiment interdicted all further experiments.

Of the patients intrusted to the care of Dr. Klein, twelve were dismissed cured in fifteen days; one had to be treated fourteen days longer for a subsequent eruption; and the last, with purulent itch, was cured in seven weeks. They were all treated with kali soap, according to Graff's method, which, during the last two and a half years, has been proved, in the garrison hospital at Stuttgardt, to be safer and more rapidly productive of a good effect than any other. This soap (which is, properly speaking, an improvement on Graff's,) is composed of kali and train-oil, and is not, like his, of such a stimulating nature as frequently to produce a fresh eruption, after having cured the original complaint.

*Wirtemberg Corrispondenz-blatt.* Band iv.

IV. *On Solutions of Caustic in the Treatment of Scabies, in all its forms.* By M. MALAPERT. Report of MM. ALIBERT, BIETT, and BOUSQUET.

M. Malapert regards the want of cleanliness of the common method employed in the treatment of scabies, and its frequent relapse after such treatment, as strong reasons for the employment of other means, and he has had recourse to the old method of the employment of caustics in this eruption. He says that "any dissolved caustic, which, without altering the healthy portion of the skin, can act directly upon the disease, is capable of curing scabies." The best solvent is water. The number of applications which should be employed must depend on the sensibility of the individual. The comparative value of caustics is stated in the following list:—Caustic potass, soda, corrosive sublimate, sulphuric acid, subcarbonate of potass. The effect of sulphuric acid is most rapid, twelve days being the average time required for cure; but, of nineteen cases, there were four relapses. The corrosive sublimate is the quickest and most certain in its effects; it cures the disease in fifteen days; the proportions employed are, twelve grains to one ounce of water. Potass and soda require another day to affect a cure. The subcarbonate of potass is the slowest in its effects.

*Bulletin de l'Academie Royale de Mèdecine,* No. 1. 1836.

---

*Case of an entire Division of the Windpipe, with an almost complete Division of the Gullet, cured.* By Dr. MICHAELSEN, of Meldorf.

IT chanced to Dr. Michaelsen, during the latter months of his academical career, to witness the treatment of a case of rare occurrence, in which both the larynx and pharynx were completely divided. This patient was under the care of the late Professor Lueders, in February, 1826; and a report of the case, which was treated successfully, will be found in Gräfe and Walther's Journal, band xiii. St. ii. A similar case occurred to Dr. Michaelsen himself in 1830, of which the following is an account.

M. B., a native of Schenefeld, thirty-five years of age, had committed a burglary, and, on being discovered and pursued, he determined on committing suicide. According to his own account, the attempt was made with a common pocket knife, not of large size, and straight and blunt; he held it in his left hand, and drew it thrice across his neck; and he was in the act of repeating the cut when he was seized and prevented.

This occurred on the 30th of June, 1830, at three o'clock in the afternoon. He lost a large quantity of blood, and fainted, and remained without aid for more than three hours. An attempt was then made to relieve the choking paroxysms by the exhibition of stimulants, such as wine, &c.; and in this way eighteen hours were consumed before medical aid was procured.

On the 1st of July, at mid-day, the man was seen for the first time by Dr. Michaelsen. He had by this time rallied considerably, and breathed through the wound. He expressed himself principally by signs, although he was enabled to articulate a few unintelligible words, when the edges of the wound were approximated by throwing the head forwards. His pulse was regular, and no fever was present; he was able to stand and walk about, but suffered much from a sense of faintness, and expressed a craving for food.

The state of the wounds was as follows:—Externally, the wound of the soft parts extended about four inches in front of the windpipe. The larynx was entirely divided, the inferior portion of the thyroid cartilage being severed from the glottis and epiglottis; and these latter, together with the superior cornua and arytenoid cartilages, remaining in connexion with the tongue. The divided portion of the larynx which remained attached to the trachea was drawn downwards, and corresponded to the inferior margin of the external wound. The pharynx was divided through nearly the whole of its circumference, about a quarter of an inch of its posterior wall alone remaining unsevered.

After a careful cleansing of the wound, its edges were brought together, and kept in apposition by means of the bloody suture; the adaptation being so arranged as to allow of the free exit of any discharge; and, as respiration by the nose and mouth was now perfectly re-established, there was no scruple in leaving the sutures. A linen compress dipped in oil was then applied by way of dressing; and the head being approximated to the breast, was fixed in this position by means of bandages made fast to the nightcap, and carried in a crucial direction over the chest. The patient was then so placed that he was obliged to retain the same fixed position, and two attendants were left in charge of him. The only nourishment allowed him was boiled milk sweetened, which he was enabled to swallow with tolerable facility, unless he took it in too large quantity, when it flowed into the larynx and produced a suffocating cough, subsequently making its escape by the wound. All other food was strictly forbidden, and the attendants were ordered to keep him quiet and silent, and not to neglect a proper ventilation of the room.

On July 3d, the patient appeared comfortable, but, as a violent fit of coughing had occurred, it was thought advisable to examine the state of the wound. On removal of the dressing, it was found that two of the sutures were torn out, and that a quantity of paste was lying in the wound. This was explained by the attendants, who confessed to having supplied the patient with some hard dumplings and pancakes. After carefully cleansing the wound, which did not now gape to more than half its original extent, the edges were again approximated by two strong sutures, and, the bandages being reapplied as before, the attendants received strict orders to confine the patient to milk. There was no febrile excitement, and the pulse was natural. Next day, the sutures remained undisturbed, and the healing was proceeding favorably, there being a thick tenacious discharge from the wound. The man was able to speak, and swallowed without difficulty. Pulse natural; appetite good, bowels regular; slept well. On the 8th, all but the anterior part of the wound was healed. The patient, however, complained much of debility, and coughed with considerable expectoration of mucus. Two of the sutures had again given way, but were not renewed, because position now appeared sufficient to keep the margins of the wound in contact. On the 23d, the wound was quite healed; but the patient complained of a pricking pain in the right side of the throat. For this a few leeches were applied, and subsequently a blister; and on the 25th, (within a month of the receipt of the injury,) the cure was complete.

In concluding the narration of this case, Dr. Michaelsen directs the attention to many points of interest connected with it. He remarks upon the singular fact that the blood-vessels and nerves of the neck, particularly those of the right side, should have been uninjured; and observes, that the present instance, in which a straight knife was employed, combats the opinion held by some that a wound of this nature could only be made by a curved knife. It is also shown by this case, as Dr. M. observes, that an entire division of the larynx, and an all but complete division of the pharynx, without simultaneous injury of the great blood-vessels or nerves of the neck, must not be classed among those injuries which of necessity produce death, as was taught by the older practitioners. The cure in this instance was singularly perfect, no inconvenience of any sort remaining; and this is the more remarkable from the length of time that elapsed before any medical aid was procured, and in spite of the subsequent interference with the curative process, occasioned by moving the patient, and subjecting him to an examination in which he was forced to exert himself in answering questions, &c. Altogether it is a rare example of the restorative powers of nature, almost unaided by art.

*Pfaff's Mittheilungen, &c.* Heft xi.-xii. 1836.

